# Insecticidal Activity of *Eupatorium fortunei* Essential Oil Against *Schizaphis graminum* and Its Effects on Detoxification Enzymes

**DOI:** 10.3390/insects16111141

**Published:** 2025-11-07

**Authors:** Guochang Wang, Dongbiao Lü, Xing Ge, Ziyue Zhang, Fanning Meng, Liuping Chen, Kassen Kuanysh, Xinan Li, Baizhong Zhang, Sarsekova Dani, Hongliang Wang

**Affiliations:** 1 Henan Province Engineering Research Center of Biological Pesticide & Fertilizer Development and Synergistic Application, College of Plant Protection and Environment, Henan Institute of Science and Technology, Xinxiang 453003, China; wgchslbh@163.com (G.W.); ge_xing_22@126.com (X.G.); zzy_94983@163.com (Z.Z.); luckymfn@163.com (F.M.); chenliupinghist@163.com (L.C.); lixinan2019@126.com (X.L.); baizhongok@163.com (B.Z.); 2 Plant Biotechnology Center, Kazakh National Agrarian Research University, Almaty 050040, Kazakhstan; kassenkuanysh12@gmail.com; 3 Hebi Institute of Engineering and Technology, Henan Polytechnic University, Hebi 458030, China; 4 Faculty of Forestry and Land Resources, Kazakh National Agrarian Research University, Almaty 050040, Kazakhstan

**Keywords:** *Schizaphis graminum*, essential oils, insecticidal activity, enzymatic activity

## Abstract

*Schizaphis graminum* is the most important pest of wheat, and its feeding harm not only affects photosynthesis but also causes diseases, posing a serious threat to wheat production. We measured the contact killing and fumigation activities, population changes, enzyme activities, and nanoemulsion physicochemical property of *Eupatorium fortune* essential oil (EFEO) and pyrethrin against *S. graminum* and its natural enemy (*Harmonia axyridis*). Toxic effects of EFEO and pyrethrin increased with increasing concentration. After 24 h of contact killing, the LD_50_ of *S. graminum* were 9.23 and 0.02 μg/head, respectively. *Harmonia axyridis* were 36.71 and 0.07 μg/head. The fumigation of EFEO LD_50_ on *S. graminum* was 9.779 mg/L (y = 2.88x − 2.852). EFEO contains 28 chemical components, including l-Caryophyllene, Lily aldehyde, α-Terpineol, and Cineole. EFEO LD_50_ significantly reduced the adult longevity, nymph production, and nymph production duration of the F0 *S. graminum*, while there were no significant differences in various parameters of offspring. The activity of acetylcholinesterase, glutathione-S-transferase, and carboxylesterase is significantly affected. The population decline rate of EFEO nanoemulsion is significantly higher than that of EFEO. This research aims to clarify the toxic effect of EFEO on *S. graminum*, providing a scientific basis for developing new plant essential oil preparations and nano preparations.

## Simple Summary

*Schizaphis graminum* is the most significant pest of wheat, causing severe damage to leaves, stems, and young ears. Its feeding not only disrupts photosynthesis but also facilitates the spread of diseases, resulting in significant yield losses. In this study, we measured the contact killing and fumigation activities of *Eupatorium fortune* essential oil (EFEO) and pyrethrin against *S. graminum* and its natural enemy, *Harmonia axyridis*. The main components of EFEO were identified, and its contact killing activity was confirmed. The sublethal effects of EFEO (LD_50_) on the population changes (F0 and F1 generations) of *S. graminum* and the activities of three detoxification enzymes (acetylcholinesterase, glutathione-S-transferase, and carboxylesterase). We also formulated EFEO nanoemulsions, characterized their physicochemical properties, and tested their impact on *S. graminum* population decline rates. This research aims to clarify the toxic effect and control efficacy of EFEO, providing a scientific basis for developing new plant essential oil preparations and nano preparations.

## 1. Introduction

*Schizaphis graminum* (Rondani) (Hemiptera: Aphididae) is one of the most common and destructive pests in wheat production. Both adults and nymphs feed on leaves and other plant parts by sucking sap and secreting honeydew. This feeding not only disrupts photosynthesis but also promotes the growth and transmission of bacterial and viral pathogens, leading to wheat diseases and posing a serious threat to crop health and yield [1,2]. *S. graminum* mainly reproduces by parthenogenesis, with a short reproductive cycle. There are 20 to 30 generations each year, and the number of generations varies by region. It has the characteristics of migratory and diffusive spread, as well as a wide occurrence area and high degree of damage [3]. The harm of aphids not only directly affects wheat yield but also poses a potential threat to food security [3]. The long-term and excessive use of chemical pesticides has led to a series of complex and far-reaching negative effects [4], including the “3R” issues, environmental pollution and ecological damage, food safety concerns, and risks to human health [5,6]. Based on the sustainable and balanced development of the agricultural environment and the pursuit of green and safe quality, it is urgent to develop and apply green and efficient substitutes to avoid or mitigate the impact of chemical pesticides on the ecological environment. Exploring and developing substitutes for chemical pesticides has become an important direction in current agricultural scientific research.

*Eupatorium fortune* (Turcz.) (Asterales: Asteraceae) is widely distributed in China. Modern pharmacological research has shown that it possesses significant antibacterial, antiviral, and anti-inflammatory properties [7]. Plant essential oils are secondary metabolites extracted from various plant fruits, leaves, flowers, and roots. The main constituents of plant essential oils include terpenes and ketones, which are low molecular weight and highly volatile compounds [8]. Plant essential oils and their components have demonstrated diverse biological activities, including contact killing, fumigation, behavioral regulation, and growth inhibition against insect pests. Compared with synthetic insecticides, they generally exhibit lower toxicity toward non-target pests (such as natural enemies) and are often more environmentally compatible [9,10]. Czerniewicz et al. tested the activities of four essential oils from the Asteraceae plants (*Achillea millefolium*, *Santolina chamaecyparissus*, *Tagetes patula,* and *Tanacetum vulgare*) on *Myzus persicae* and found that the essential oils from *S. chamaecyparissus* and *A. millefolium* exhibited the strongest toxicities, followed by those from *T. vulgare* and *T. patula* [11]. Czerniewicz et al. conducted tests on the toxic effects of four essential oils on *Rhopalosiphum padi*, as well as their influence on enzymes related to insect protein and sugar metabolism, such as trypsin, pepsin, and α- and β-glucosidase activities [12]. Akbari et al. discovered that the essential oil of *A. millefolium* has a very high toxicity towards *Aphis gossypii* and also affects the reproductive capacity of the population [13]. Ortiz de Elguea-Culebras et al. discovered that the essential oil of *S. chamaecyparissus* has a strong antifeedant activity against *R. padi* [14]. The essential oils of Asteraceae plants have remarkable effects in controlling aphids and possess great potential for development and application. Tabanca et al. demonstrated that *Eupatorium capillifolium* essential oil exhibits significant repellent activity against *Aedes aegypti* [15]. Guerreiro et al. revealed the fumigation toxicity of *Eupatorium buniifolium* essential oil against *Triatoma infestans* [16]. Sosa et al. studied the antifeedant activity of essential oils from *Eupatorium* species (*E. buniifolium*, *Eupatorium inulaefolium*, *Eupatorium arnotii*, and *Eupatorium viscidum*) against *M. persicae* and *R. padi* [17]. Zhang et al. reported the fumigation toxicity of *E. fortunei* essential oil against *Tribolium castaneum* and *Lasioderma serricorne* [18]. Based on the effects of *Eupatorium* species essential oil and *E. fortunei* essential oil on pests, we could explore the impact of *E. fortunei* essential oil on *S. graminum*. Plant essential oils have become the best materials for developing botanical pesticides due to their low toxicity and pests not easily developing resistance.

The complex chemical composition of plant essential oils underlies their multiple modes of action, including contact killing activity, gastric toxicity, inhalation activity, fumigation activity, feeding deterrence, and behavioral regulation [19]. Plant essential oils have a contact toxicity effect on pests. When pests come into contact with essential oils, they experience extreme dizziness, excitement, convulsions, and other symptoms [20]. Some essential oils have the ability to destroy the structure of insect body walls, dissolve cuticula, facilitate the penetration of pesticides, and promote the penetration, absorption, transport, and entry of pesticide components into stomata [20]. For instance, Wang et al. found that the higher the concentration of *Cercidiphyllum japonicum* essential oil, the stronger the contact killing effect on *M. persicae* [21]. Liu et al. found that four essential oils (*Ocimum basilicum*, *Agastache rugosa*, *Sabina vulgaris*, and *Mentha canadensis*) have significant effects on the activities of acetylcholinesterase (AChE), glutathione-S-transferase (GSTs), and carboxylesterase (CarE) activities in *Aphis* sp., leading to a significant contact killing effect [22]. The fumigation activity of essential oils may be associated with their interference with the insect nervous system and key detoxification enzymes (such as AChE, GSTs, CarE, etc.) [23]. Martín et al. found that after spraying plants with a 0.2% nanoemulsion of farnesol, the average duration of non-foraging activities by *M. persicae* cotton aphids increased, and the population growth rate slowed down [24]. Mondal et al. analyzed the characteristics of the nanoemulsions of the oil and carvone and evaluated its efficacy against *Rhopalosiphum maidis* and *Sitobion avenae* [25]. They concluded that the nanoemulsion of carvone showed a significant increase in the mortality rate of aphids and the inhibition activity of acetylcholinesterase within 24 h. Abdelaal et al. prepared nanoemulsions of four essential oils (*Basilicum ocimum*, *Cuminum cyminum*, *Origanum marjorana*, and *Matricaria chamomilla*) and found that the nanoemulsions exhibited considerable toxicity against cowpea aphids and significantly altered the activities of aphid acetylcholine esterase, alkaline phosphatase, β-esterase, glutathione S-transferase (GST), and mixed function oxidase (MFO) [26]. Overall, nanoemulsions are a potential tool for controlling aphids. Plant essential oils, with their unique biological activity and diverse modes of action, have shown great potential in sustainable pest management and are expected to play a key role in future green agricultural production systems.

In this study, we measured the contact killing activity of *Eupatorium fortune* essential oil (EFEO) and pyrethrin against *S. graminum*, the fumigation activity of EFEO against *S. graminum*, examined the contact killing activity of the main compounds of EFEO against *S. graminum*, the impact of EFEO LD_50_ on the population change and enzyme activity of *S. graminum*, the safety evaluation of EFEO, and the characterization of the physical and chemical properties of EFEO nanoemulsion. Clarify the control effect of EFEO on *S. graminum*. This study delves into the chemical composition and mechanism of EFEO and develops plant essential oil formulations suitable for wheat aphid control, providing a scientific basis for the development of new green pesticides.

## 2. Materials and Methods

### 2.1. Insect Culture and Reagents

Laboratory trials were carried out at the Henan Institute of Science and Technology, Xinxiang, Henan, China. *S. graminum* and *H. axyridis* were all collected in the field in the Xinxiang comprehensive experimental base of the Chinese Academy of Agricultural Sciences (Xinxiang, China). *S. graminum* and *H. axyridis* were raised in insect rearing chambers with meshed cages (50 × 50 × 50 cm^3^). *S. graminum* feed on wheat seedlings, while *H. axyridis* feed on *S. graminum*. The population was kept at 20 ± 1 °C with 70 ± 5% relative humidity under a 16:8 h light/dark [27].

*E. fortune* essential oil (EFEO, Jian Huantianbao Herbs Biological Products Factory, Ji’an, China), Pyrethrin (50% active ingredient, *w*/*w*, Beijing Kingbo Biotech Co., Ltd., Beijing, China), n-Hexane (AR, Tianjin De’en Chemical Reagent Co., Ltd., Tianjin, China), l-Caryophyllene (AR, Beijing J&K Scientific Ltd., Beijing, China), Cineole (AR, Beijing J&K Scientific Ltd., Beijing, China), α-Terpineol (AR, Beijing J&K Scientific Ltd., Beijing, China), Lily aldehyde (AR, Shanghai Kasei Industrial Development Co., Ltd., Shanghai, China).

### 2.2. Contact Killing Activity of EFEO and Pyrethrin Against S. graminum

The contact killing activity was determined by the drop method [28]. EFEO was diluted with n-hexane to five concentrations of 4.60, 7.80, 11.00, 15.50, and 19.00 μg/μL, while pyrethrin was diluted to 0.0035, 0.0089, 0.0222, 0.0555, and 0.1387 μg/μL. In petri dishes (D = 9.00 cm) containing wheat leaves, thirty third-instar aphids were placed. One μL of each of the above five concentrations of EFEO and pyrethrin solution was dropped onto the dorsal plates of the aphids, and the n-hexane solution was used as the control group. The samples were placed in a climate chamber for observation. The mortality of aphids was recorded after 4, 8, and 24 h of treatment. The mortality rate and corrected mortality rate were calculated according to the method of Ji et al. [29]. Each concentration was tested with three biological replicates.

### 2.3. Fumigation Activity of EFEO Against S. graminum

Place fresh wheat leaves in a 50 mL triangular flask, add 20 third-instar aphids, and hang a 2 cm × 3 cm filter paper strip at the bottle mouth. Dilute the EFEO with n-hexane to five concentrations and take 50 μL of each solution to the filter paper strip. The fumigation concentrations were 5.508, 8.262, 11.016, 13.77, and 16.524 mg/L. The n-hexane solution was the control group. After completion, quickly close the bottle cap, record the death of aphids after 24 h of treatment, and calculate the mortality rate and corrected mortality rate. Each concentration was tested with three biological replicates.

### 2.4. Contact Killing Activity of the Main Compounds of EFEO Against S. graminum

A total of 100 μL EFEO was added to 900 μL of n-hexane to determine the composition of EFEO. Through gas chromatography-mass spectrometry (GC-MS) detection, the GC conditions were as follows: The chromatographic column was a DB-35 capillary column (30 m × 250 μm × 0.25 μm); the injection port temperature was 220 °C, the initial temperature was 50 °C, maintained for 2 min, then increased at a rate of 10 °C/min to 180 °C, maintained for 5 min, then increased at a rate of 20 °C/min to 240 °C, maintained for 5 min; the injection volume was one μL. MS was at 150 °C for the quadrupole and 230 °C for the ion source. The retention indices were determined in relation to a homologous series of n-alkanes (C7–C40) under the same operating conditions. The components were identified by the National Institute of Standards and Technology mass spectral library (NIST 14.L) and confirmed by comparing the Kovats Indices (KI) and comparison with authentic standards (when available) [30]. Relative percentages of the individual components of the EO were quantified on the basis of the peak area, which was integrated in the analysis program. The contact killing activity of the main compounds of EFEO was screened through the above drop method.

### 2.5. Sublethal Effects of EFEO (LD_50_) on the Population of S. graminum

One hundred third-instar aphids were introduced into fresh wheat seedlings and raised in an artificial climate incubator for 24 h. Then, nymph aphids were selected and continued to be raised until the third-instar. The test organisms (F0) were treated with EFEO LD_50_ using the drop method according to the biological assay protocol. The n-hexane treatment was used as the control group. After 24 h, the mortality rate of the aphids was recorded. This was used for the subsequent sub-lethal effect experiment of EFEO. Randomly select 75 healthy and consistent larvae and place them in petri dishes for single-head single-plant cultivation. The wheat seedlings were replaced every three days, and the survival rate, mortality rate, and the number of offspring aphids were recorded daily. After counting the newly produced offspring aphids each day, they were removed from the wheat seedlings until the adult aphids died. The first generation (F1) and the second generation (F2) were the nymph aphids produced by F0 and F1, respectively. A total of 75 individuals were randomly selected for single-head single-plant cultivation and observed and recorded using the same method. All the obtained data were used to establish the life table of *S. graminum* [29].

### 2.6. EFEO LD_50_ on the Enzyme Activity of S. graminum

Collect the aphids treated with EFEO LD_50_ at 4, 8, 12, 24, and 48 h, and conduct enzyme activity assays. A total of 50 third-instar aphids were used as a sample, and each sample has three biological replicates. The sample pretreatment is carried out according to the method of Wang et al. [31]. The determination of protein concentration, AChE, GSTs, and CarE is conducted according to the corresponding activity assay kits (Jiancheng, Nanjing, China).

### 2.7. Safety Evaluation of EFEO

The contact killing activity of EFEO and pyrethrin on *H. axyridis*. The measurement was conducted using the drop method. A second-instar larvae was separately dripped with 2.90, 5.70, 11.50, 23.00, and 45.90 μg/head of EFEO solution and with 0.0444, 0.0666, 0.0887, 0.1109, and 0.1331 μg/head of pyrethrin solution. The mortality was recorded at 4, 8, 24, and 48 h after treatment, and the mortality rate and corrected mortality rate were calculated. Thirty *H. axyridis* were used as a sample, with three biological replicates at each concentration.

### 2.8. Preparation and Physicochemical Property Characterization of EFEO Nanoemulsion

Using chitosan as the nanocarrier to prepare EFEO. Chitosan was added to a 1% acetic acid aqueous solution to prepare 50 mL chitosan solution. A total of 3 mL of Tween-80 was added, and the mixture was stirred at 40 °C overnight to obtain a homogeneous mixture. A total of 5 mL of EFEO was added to obtain a peppermint nanoemulsion. The emulsion was subjected to Fourier Transform Infrared Spectroscopy (FTIR) detection to evaluate the combination of EFEO and the nanocarrier.

Toxicological biological assay. Wheat was planted in pots (D = 12.5 cm). At the 2–3 leaf stage of wheat, 50 third-instar aphids were introduced. After colonization, each treatment was sprayed once using an ultra-fine sprayer (working pressure 0.2–0.4 MPa) with 2 mL of EFEO nanoemulsion (applied via ultra-fine spraying to cover every leaf and the entire pot area). Following treatment, the pots were individually maintained in growth chambers (20 ± 1 °C, 70 ± 5% RH, and 16:8 h L:D photoperiod), the number of aphids was recorded at 1, 3, and 5 d post-application, and the reduction rate of the pest population and the corrected control effect were calculated [29]. A total of 10% EFEO was used as the treatment group, and the control group was without EFEO. Each treatment was replicated three times.

### 2.9. Data Analysis and Statistics

Data were analyzed using SPSS Statistics 26.0 (https://www.ibm.com/products/spss-statistics, accessed on 15 June 2025). Probit analysis was conducted to estimate LD_50_ and LC_50_ values with their corresponding 95% confidence limits. Pairwise comparison was performed using Student’s *t*-test analysis. One-way ANOVA and Tukey’s HSD test were used for comparisons of greater than two groups with the following notations: **, significant difference at *p* < 0.01; *, significant difference at *p* < 0.05; ns, no significant difference.

## 3. Results

### 3.1. Contact Killing Effect of EFEO Against S. graminum

The corrected mortality rate of aphids increased with the increase of the exposure dose of EFEO. The mortality rate at 4 h was from 32.33 ± 2.33 to 90.00 ± 1.73, at 8 h from 30.30 ± 1.58 to 86.76 ± 2.03, and at 24 h from 28.67 ± 2.96 to 80.00 ± 1.73 (Table 1). EFEO showed a significant contact killing effect within a short period of time, 4 and 8 h (*p* < 0.05), and its insecticidal activity remained relatively stable within 24 h, indicating that EFEO has contact killing activity against aphids. The toxicity regression curves of the biological activity of EFEO on different treatment times of *S. graminum* were analyzed. It was concluded that after 4, 8, and 24 h, the median lethal dose (LD_50_) of EFEO against aphids was 8.09, 8.13, and 9.23 μg/head, respectively (Table 2).

### 3.2. Contact Killing Effect of Pyrethrin Against S. graminum

The corrected mortality rate of aphids increased with the increase of pyrethrin exposure dose. The mortality rate at 4 h was from 9.57 ± 0.08 to 60.00 ± 3.33, at 8 h from 13.16 ± 1.68 to 80.70 ± 3.04, and at 24 h from 20.18 ± 1.68 to 90.35 ± 1.68 (*p* < 0.05) (Table 3). At 4 h, the contact killing effect at each concentration was relatively low, but as time progressed to 8 and 24 h, the contact killing effect significantly improved. The toxicity regression curve of the biological activity of pyrethrin on aphids was obtained. After 4, 8, and 24 h, the LD_50_ of pyrethrin against aphids were 0.12, 0.04, and 0.02 μg/head, respectively (Table 4).

### 3.3. Fumigation Effect of EFEO Against S. graminum

As the concentration of EFEO increased, the corrected mortality rate of the aphids significantly increased. At 5.508 mg/L, the corrected mortality rate was 27.12%, whereas at 16.524 mg/L, it reached 80.51%. EFEO has a fumigating effect against *S. graminum* (Figure S1). The toxicity regression curve of the fumigating of EFEO on aphids was calculated, and it was found that at 24 h, the LD_50_ of EFEO was 9.779 mg/L (Table 5).

### 3.4. Contact Killing Effect of Main Compounds in EFEO Against S. graminum

After identifying the chemical components of EFEO, 28 chemical components were obtained (Table S1). Among them, the four main compounds with relatively high contents were l-Caryophyllene, Lily aldehyde, α-Terpineol, and Cineole, with their contents being 44.66%, 22.36%, 12.17%, and 10.74%, respectively. The contact killing effect of the four compounds against *S. graminum* gradually increased with the increase of concentration and time (Table 6). The toxicity regression curves of the four compounds were analyzed, and the LD_50_ of the four compounds after treatment for 24 h were obtained as follows: Lily aldehyde was 3.96, α-Terpineol was 6.27, l-Caryophyllene was 9.16, and Cineole was 12.31 μg/head (Table 7). According to Table 2, the contact killing toxicity LD_50_ of EFEO against aphids was 9.23 μg/head. Meanwhile, Lily aldehyde and α-Terpineol exhibit stronger aphid-killing effects at the same concentration.

### 3.5. Inhibitory Effect of EFEO LD_50_ on the Population of S. graminum

**Figure 1:**
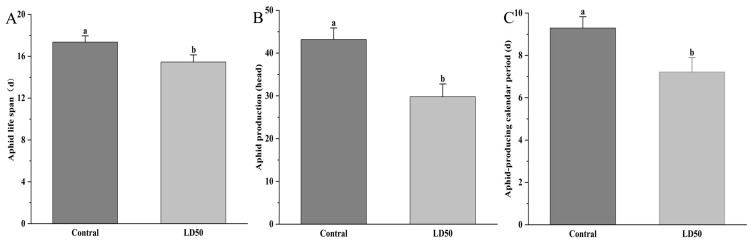
Effects of EFEO LD_50_ on the adult longevity (**A**), nymph production (**B**), and nymph production duration (**C**) of F_0_ of *S. graminum.* Different lowercase letters indicate significant differences (*p* < 0.05).

Treatment with EFEO at the LD_50_ concentration significantly affected the F0 generation of *S. graminum*. Adult longevity treated was 15.54 d, significantly shorter than that of the control group was 17.36 d (*p* < 0.05); the number of female aphids producing nymphs was 29.80, significantly lower than that of the control group, which was 43.17 (*p* < 0.05); the nymph production duration was 7.21 d, significantly shorter than that of the control group, which was 9.29 d (*p* < 0.05). Overall, EFEO at the LD_50_ concentration significantly reduced adult longevity, nymph production, and nymph production duration of the F0 generation of *S. graminum* (*p* < 0.05) (Figure 1).

**Figure 2:**
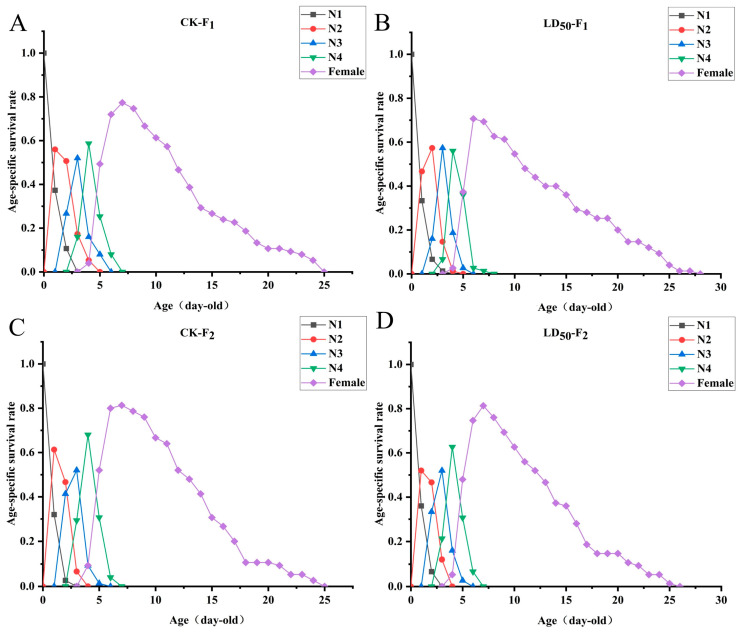
Effects of EFEO LD_50_ on the age-stage survival rate (*s_xj_*) of F_1_ and F_2_ of *S. graminum*. (**A**) CK to F1. (**B**) LD_50_ to F1. (**C**) CK to F2. (**D**) LD_50_ to F2.

**Figure 3:**
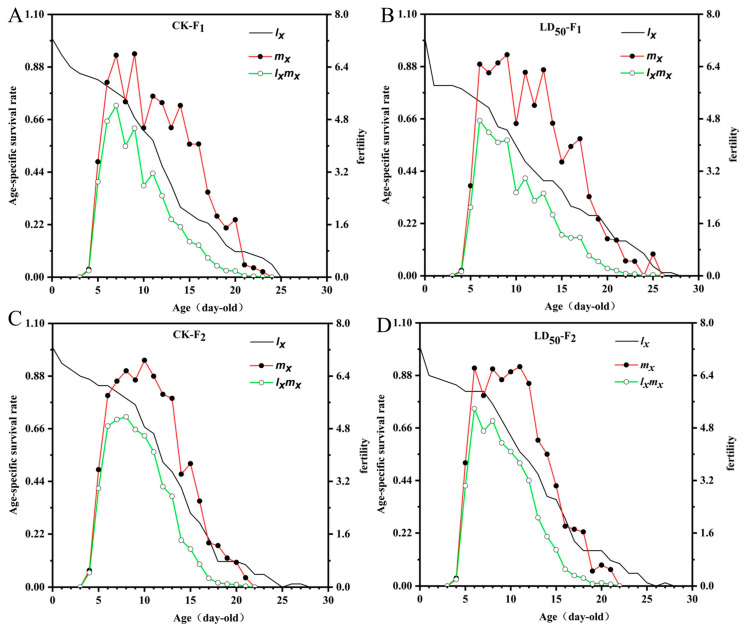
Effects of EFEO LD_50_ on age-specific survival (*l_x_*), age-specific fecundity (*m_x_*) and population age-specific reproductive values (*l_x_m_x_*) of F_1_ and F_2_ of *S*. *graminum.* (**A**) CK to F1. (**B**) LD_50_ to F1. (**C**) CK to F2. (**D**) LD_50_ to F2.

EFEO treatment at the LD_50_ concentration had no significant effects on the developmental duration, preadult period, adult longevity, total longevity, adult pre-oviposition (APOP), total pre-oviposition period (TPOP), aphid production, and fecundity of the F1 and F2 generations of *S. graminum* (*p* > 0.05) (Figure 2 and Table S2). Similarly, EFEO had no significant effect on innate rate of increase (*r_m_*), finite rate of increase (*λ*), net reproductive rate (*R*_0_), or mean generation period (*T*) (*p* > 0.05) (Figure 3 and Table S3).

### 3.6. EFEO LD_50_ Impact on the Enzyme Activity of S. graminum

**Figure 4:**
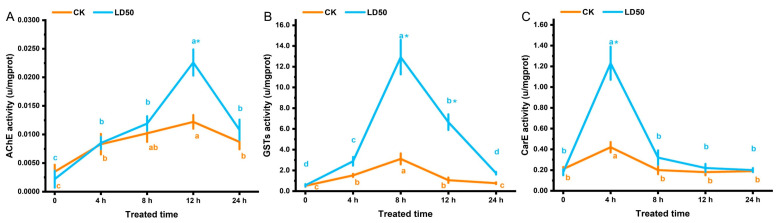
Changes in the enzyme activity of *S. graminum* at different time points following EFEO treatment at the LD_50_ concentration. (**A**): AChE; (**B**): GSTs; (**C**): CarE. Different lowercase letters indicate significant differences between different time points (*p* < 0.05; ANOVA with Tukey’s HSD test), and * indicates significant differences between the treatment group and the control group at the same time point (*p* < 0.05; *t*-test).

EFEO treatment at the LD_50_ concentration significantly affected enzyme activities in *S. graminum* over time (*p* < 0.05). AChE activity increased slowly from 0 to 8 h, then sharply increased to the maximum value of 0.0226 ± 0.0023 at 12 h, which was significantly higher than other times, and then decreased sharply at 24 h (*p* < 0.05). The control group also reached the maximum value at 12 h, but the treatment group was significantly greater than the control group, and there was no significant difference at other times (*p* > 0.05) (Figure 4A). GST activity increased slowly from 0 to 4 h, then sharply increased to the maximum value of 12.92 ± 1.67 at 8 h, which was significantly higher than other times (*p* < 0.05), and then decreased sharply at 12 and 24 h. The control group also reached the maximum value at 8 h, but the 8 and 12 h treatment groups were significantly greater than the control group, and there was no significant difference at other times (*p* > 0.05) (Figure 4B). CarE activity sharply increased to the maximum value of 1.23 ± 0.16 from 0 to 4 h, which was significantly higher than other times (*p* < 0.05), and then decreased sharply after 8 h and basically stabilized, with no significant difference (*p* > 0.05). The control group also reached the maximum value at 4 h, but the treatment group was significantly greater than the control group (*p* < 0.05), and there was no significant difference at other times (*p* > 0.05) (Figure 4C).

### 3.7. Safety Verification of EFEO

The contact killing activity of EFEO and pyrethrin on *H. axyridis* gradually increases with the increase of concentration and time (*p* < 0.05). The mortality rate of EFEO at 4 h was from 0.00 ± 0.00 to 52.50 ± 4.80, at 8 h from 00.00 ± 0.00 to 62.50 ± 8.50, at 24 h from 12.50 ± 2.50 to 65.00 ± 9.60, and at 48 h from 22.50 ± 4.80 to 67.50 ± 7.50 (Table 8). The toxicity regression curves of the biological activity of EFEO on different treatment times of *H. axyridis* were analyzed. It was concluded that after 4, 8, 24, and 48 h, the LD_50_ of EFEO against *H. axyridis* were 44.80, 37.34, 36.71, and 26.07 μg/head, respectively (Table 9). The mortality rate of pyrethrin at 4 h was from 15.00 ± 2.90 to 77.50 ± 2.50, at 8 h from 22.50 ± 4.80 to 80.00 ± 4.10, at 24 h from 30.80 ± 2.60 to 84.60 ± 3.00, and at 48 h from 32.40 ± 2.70 to 86.50 ± 2.70 (Table 10). The toxicity regression curves of the biological activity of pyrethrin on different treatment times of *H. axyridis* were analyzed. It was concluded that after 4, 8, 24, and 48 h, the LD_50_ of EFEO against *H. axyridis* were 0.09, 0.08, 0.07, and 0.07 μg/head, respectively (Table 11).

### 3.8. The Control Efficiency of EFEO Nanoemulsion

FTIR shows that the surface-modified nanocarriers and nanoemulsion have slightly changed (Figure S2). Peaks of EFEO and chitosan appear at 1250, 1500, and 2900 cm^−1^. Indicates that the combination of EFEO and chitosan has been successful. The population decline rates of the EFEO nanoemulsion and 10% EFEO were 54.00 ± 6.11% and 32.67 ± 6.57% at 1 d, 73.33 ± 4.80% and 48.00 ± 4.16% at 3 d, and 74.67 ± 4.67% and 60.00 ± 2.31% at 5 d, respectively. The control group was below 10% (Table 12).

## 4. Discussion

At an EFEO concentration of 4.6 μg/head, the corrected mortality rate of *S. graminum* was approximately 25%, while at 17.40 μg/head, it reached 75%–90%. Indicates that high concentrations of EFEO have a good contact killing effect on *S. graminum*. Sharma et al. found that the LD_50_ of the *Murraya koenigii* essential oil for *Aphis craccivora* and *Planococcus lilacinus* were 1.29–1.38 μL/head and 2.63–3.06 μL/head, respectively [32]. Over time, some aphids have shown signs of revival. A similar phenomenon has been observed in wolfberry psyllids [10], indicating that the insecticidal effect of Perrin essential oil is not persistent but rather has a certain degree of timeliness. This recovery phenomenon is also observed in *Poratrioza sinica*, indicating that the effect of EFEO fumigation is not long-lasting but has a certain degree of timeliness [10]. Liu et al. reported that essential oils had a significant fumigation effect on adult *P. sinica* within 4 h; however, recovery occurred after 15 h [10]. The fumigation effect of EFEO against *S. graminum* was significant, with corrected mortality increasing from 27.12% at 5.508 mg/L to 80.51% at 16.524 mg/L. Similarly, Tak et al. studies showed that *Thymus mongolicus* essential oil had strong contact and fumigation effects against *Trichoplusia ni* compared with *Cymbopogon citratus* essential oil [33].

Botanical insecticides are generally considered environmentally friendly and compatible with biological pest control methods. Lami et al. compared the efficacy of five different categories of botanical insecticides and insecticidal soaps against *M. persicae*, concluding that pyrethrin-based products exhibited the highest aphid mortality [34]. Xu et al. evaluated ten botanical insecticides against *R. padi*, finding rotenone and pyrethrin to be the most toxic, with efficacy rivaling conventional insecticides. Following 24 h exposure to sublethal concentrations (LC_10_, LC_30_) of either compound, the longevity and fecundity of F_0_ adults were significantly reduced compared to the control group [35]. Our results confirm that pyrethrins are more effective against *S. graminum* than the essential oil, aligning with findings from multiple studies.

GC-MS analysis identified l-Caryophyllene, Lily aldehyde, α-Terpineol, and Cineole as the main components in EFEO. The LD_50_ values of Lily aldehyde and α-Terpineol are 3.96 μg/head and 6.27 μg/head, respectively. The contact killing activity was significantly higher than that of EFEO, indicating their strong insecticidal potential and prospect of developing them into botanical insecticides. Liu et al. found that *β*-Caryophyllene has high toxicity against *A. gossypii* [36].

Due to the spatial distribution and continuous degradation of insecticides in the field, insect populations are often exposed to low concentrations of insecticides, and these sublethal exposures can lead to changes in insect population dynamics [37]. EFEO LD_50_ significantly reduced adult longevity, nymph production, and nymph production duration in the F0 generation of *S. graminum*. Sublethal effects have been widely documented in other studies. Shen et al. found that the sublethal concentration LC_40_ of azadirachtin, matrine, and rotenone significantly prolonged the second-instar nymph and pupal stages of thrips and inhibited pupation rate, emergence rate, single female egg production, and egg hatching rate [38]. Wang et al. found that LC_10_ and LC_25_ concentrations of sulfoxaflo significantly reduced adult longevity and aphid yield of the F0 generation of *M. persicae* [39]. Tang et al. observed that sublethal doses of imidacloprid significantly enhanced the reproductive capacity and intrinsic rate of increase *r_m_* in the F_1_ generation of *M. persicae* [40]. Yu et al. showed that the sublethal concentration LC_15_ of chitosan reduced adult longevity and F_1_ generation reproductive capacity of wheat aphids [41]. The effects of insecticides on pests vary depending on their type and mode of action. In the studies mentioned above, some pesticides affected both the parental generation (F0) and the offspring (F1), while others only impacted the F0 generation. This study found that the LD_50_ dose of EFEO significantly reduced the adult longevity, fecundity, and reproductive period of the F0 generation of *S. graminum*, while showing no effects on the subsequent generations (F1 and F2), indicating that it does not promote population growth in *S. graminum*.

Graham et al. found that carbamate insecticides significantly reduced the activity of AChE in *A. gossypii* [42]. Gao et al. also found that omethoate and pirimicarb exerted strong inhibitory effects on AChE activity in *A. gossypii* [42]. Zhang et al. examined the effects of four pyrethrin insecticides on GST activity in *A. sp*. and found that low concentrations of pyrethrin activated GSTs, whereas higher concentrations inhibited their activity. Fenpropathrin showed the strongest induction, with GSTs activity increasing by up to 249.96% [43]. Since GSTs play a key role in detoxification in insects, reduced GST activity can disrupt normal detoxification metabolism in aphids [43]. Chen et al. demonstrated that clothianidin significantly inhibited CarE activity in *A. gossypii*, whereas acetamiprid exhibited relatively weak inhibition of aphid CarE activity in vitro [44]. Zhang et al. treated *M. persicae* with Clothianidin LC_15_ and LC_30_, resulting in a significant increase in CarE activity and a significant induction of activation [45]. The results of the present study are largely consistent with these previous findings.

The EFEO LD_50_ against *H. axyridis* at 24 h was 36.71 μg/head, while *S. graminum* was 9.23 μg/head. *M. koenigii* essential oil LD_50_ for *Sitophilus zeamais* and *T. castaneum* were 11.41 μg/head and 20.94 μg/head, respectively [46,47]. The LD_50_ of the chloroform and n-butanol essential oil extracts of *Cyperus rotundus* for *P. lilacinus* was 7.03–11.25 μg/head [48]. The main component of *C. rotundus* essential oil, namely the aqueous fraction, has an LD_50_ of 11.68 μg/head against *Aphis craccivora*. While the n-butanol and aqueous fraction had LD_50_ of 11.25 and 11.48 μg/head against *P. lilacinus*, respectively [48]. The EFEO LD_50_ of *H. axyridis* was approximately four times higher than that against *S. graminum*. This difference indicates that it is possible to achieve effective control of *S. graminum* while minimizing negative impacts on beneficial insects such as *H. axyridis*, thereby reducing impacts on non-target organisms. Compared with other plant essential oils, EFEO exhibits relatively lower toxicity and represents a promising, safe, and effective natural insecticide. Moreover, the EFEO LD_50_ against *H. axyridis* was approximately 548 times higher than that of pyrethrin, highlighting its substantially greater safety for natural enemies. Wang et al. similarly reported that azadirachtin shows high selectivity towards target pests while exhibiting low toxicity to non-target organisms, achieving effective control against aphids with low risk to *H. axyridis* [49].

The results of this study indicate that EFEO nanopreparations have significant insecticidal activity against pests, with efficacy increasing over time. This improvement may be attributed to the gradual accumulation of nano essential oil particles within the insect body. Dong et al. prepared a metal organic framework nanopesticide (Py@ZIF-8) and demonstrated through experiments that Py@ZIF-8 achieved a control efficacy of 86.67 ± 4.56% against wheat aphids [50]. Mondal et al. developed a nanoemulsion of carvone and evaluated its efficacy against *R. maidis* and *S. avenae* [25]. The results demonstrated that the carvone nanoemulsion exhibited significant aphid control activity, with 24 h mortality (LC_50_ = 0.87–1.94 mg/mL) and acetylcholinesterase inhibitory activity (IC_50_ = 0.07–3.83 mg/mL). Choupanian et al. found that nanoemulsions of essential oil were more toxic to *T. castaneum* adults than the corresponding essential oils. After 2 d of treatment, the mortality rate in the nanoemulsion group was markedly higher than that in the essential oil group [51]. Tao et al. found that botanical pesticide nanopreparations exhibited strong control efficacy against aphids in *Murraya exotica*. After 7 d, the corrected mortality rates of aphids reached 95.9% at higher concentrations and 89.8% at low concentrations, demonstrating remarkable control effects. Based on the application rate used in our experiments, the field application rate of the nanoemulsion was calculated to be 400 L/ha, while the EFEO application rate was 40 L/ha. EFEO is a natural insecticide with low toxicity and a high safety factor, making it the safest botanical pesticide with the greatest application potential in IPM in wheat fields. Notably, nanopreparations can achieve high efficacy with relatively small application volumes, making them efficient and environmentally favorable alternative [52]. Overall, essential oil nanoemulsions represent a promising strategy for the development and application of botanical pesticides.

## 5. Conclusions

This study evaluated the contact killing and fumigation activities of EFEO and pyrethrin against *S. graminum*. After 24 h of contact killing, the LD_50_ of *S. graminum* were 9.23 and 0.02 μg/head, respectively. *H. axyridis* were 36.71 and 0.07 μg/head. The fumigation of EFEO LD_50_ on *S. graminum* was 9.779 mg/L. Sublethal exposure to EFEO LD_50_ significantly reduced the adult longevity, nymph production, and nymph production duration of the F0 *S. graminum* and significantly increased the activities of three detoxification enzymes (acetylcholinesterase, glutathione-S-transferase, and carboxylesterase). EFEO has high safety towards natural enemies (*Harmonia axyridis*). The 24 h LD_50_ is 36.71 μg/head, and it has little negative impact on beneficial insects such as natural enemies. The population decline rates of the EFEO nanoemulsion significantly increased and demonstrated excellent control efficacy. This research aims to clarify the toxic effect of EFEO, providing a scientific basis for developing new plant essential oil preparations and nano preparations.

## Figures and Tables

**Table 1:** Corrected mortality rate of EFEO against *S*. *graminum*.

Time (h)	Corrected Mortality Rate (%)				
	Concentration (μg/head)				
	4.60	7.80	11.00	14.20	17.40
4	32.33 ± 2.33 ^d^	48.67 ± 2.96 ^c^	56.67 ± 2.03 ^c^	72.33 ± 2.33 ^b^	90.00 ± 1.73 ^a^
8	30.30 ± 1.58 ^d^	46.58 ± 2.45 ^c^	56.67 ± 2.03 ^c^	69.25 ± 3.02 ^b^	86.76 ± 2.03 ^a^
24	28.67 ± 2.96 ^d^	49.00 ± 4.16 ^c^	55.56 ± 2.03 ^bc^	69.00 ± 5.86 ^ab^	80.00 ± 1.73 ^a^

Means in the same row followed by different lowercase letters differed significantly (*p* < 0.05).

**Table 2:** Contact toxicity of EFEO against *S*. *graminum*.

Time (h)	Toxicity Regression Curves	Slope	R^2^	LD_50_ (μg/head)	95% CI (μg/head)	*χ* ^2^	*p*	*df*
4	y = 2.56x − 2.33	2.56 ± 0.28	0.98	8.09	4.99–10.62	8.53	0.036	3
8	y = 2.49x − 2.27	2.49 ± 0.28	0.99	8.13	5.48–10.36	6.58	0.086	3
24	y = 2.26x − 2.18	2.26 ± 0.29	0.98	9.23	8.13–10.33	1.08	0.781	3

**Table 3:** Corrected mortality rate of pyrethrin against *S*. *graminum*.

Time (h)	Corrected Mortality Rate (%)				
	Concentration (μg/head)				
	0.0035	0.0089	0.0222	0.0555	0.1387
4	9.57 ± 0.08 ^d^	13.04 ± 1.42 ^d^	20.87 ± 1.67 ^c^	28.70 ± 2.25 ^b^	60.00 ± 3.33 ^a^
8	13.16 ± 1.68 ^d^	18.42 ± 2.21 ^d^	29.82 ± 1.43 ^c^	53.51 ± 2.21 ^b^	80.70 ± 3.04 ^a^
24	20.18 ± 1.68 ^d^	24.56 ± 3.04 ^d^	50.00 ± 4.15 ^c^	66.67 ± 3.36 ^b^	90.35 ± 1.68 ^a^

Means in the same row followed by different lowercase letters differed significantly (*p* < 0.05).

**Table 4:** Contact toxicity of pyrethrin against *S*. *graminum*.

Time (h)	Toxicity Regression Curves	Slope	R^2^	LD_50_ (μg/head)	95% CI (μg/head)	*χ* ^2^	*p*	*df*
4	y = 1.02x + 0.94	1.02 ± 0.13	0.99	0.12	0.06–1.30	7.22	0.067	3
8	y = 1.31x + 1.82	1.31 ± 0.13	0.98	0.04	0.02–0.08	6.59	0.092	3
24	y = 1.35x + 2.24	1.35 ± 0.12	0.98	0.02	0.01–0.04	6.64	0.092	3

**Table 5:** Fumigation toxicity of pyrethrin against *S*. *graminum*.

Time (h)	Toxicity Regression Curves	Slope	R^2^	LD_50_ (μg/head)	95% CI (μg/head)	*χ* ^2^	*p*	*df*
24	y = 2.88x − 2.852	2.88 ± 0.34	0.99	9.779	5.922–10.653	4.89	0.180	3

**Table 6:** Corrected mortality rate of major compounds of EFEO against *S*. *graminum*.

Treatment	Concentration (μg/head)	Corrected Mortality Rate (%)		
		4 h	8 h	24 h
l-Caryophyllene	4.5	30.25 ± 2.12 ^c^	24.37 ± 4.23 ^c^	26.89 ± 4.42 ^c^
	7.7	42.86 ± 6.29 ^c^	42.02 ± 7.05 ^b^	42.86 ± 7.64 ^b^
	10.8	59.66 ± 1.37 ^b^	59.66 ± 1.37 ^a^	59.66 ± 1.37 ^a^
	14	63.03 ± 3.07 ^b^	62.18 ± 2.87 ^a^	61.34 ± 2.91 ^a^
	17.1	78.99 ± 7.05 ^a^	72.27 ± 7.69 ^a^	70.59 ± 12.69 ^a^
Lily aldehyde	1.3	17.95 ± 4.19 ^d^	15.38 ± 3.52 ^d^	14.66 ± 3.55 ^d^
	2.9	40.17 ± 2.96 ^c^	38.46 ± 4.63 ^c^	32.76 ± 2.99 ^c^
	4.6	64.10 ± 4.30 ^b^	41.00 ± 5.31 ^b^	62.07 ± 5.08 ^b^
	6.2	69.23 ± 1.97 ^b^	68.38 ± 1.64 ^b^	62.93 ± 1.65 ^b^
	7.8	83.76 ± 1.64 ^a^	81.20 ± 2.21 ^a^	76.72 ± 3.26 ^a^
α-Terpineol	1.4	11.76 ± 0.84 ^e^	10.92 ± 1.68 ^e^	10.08 ± 1.61 ^d^
	4.7	36.97 ± 5.02 ^d^	38.66 ± 5.88 ^d^	38.66 ± 6.91 ^c^
	8	63.03 ± 4.34 ^c^	56.30 ± 7.88 ^c^	50.42 ± 7.56 ^c^
	11.2	73.95 ± 2.97 ^b^	73.11 ± 3.07 ^b^	69.75 ± 4.12 ^b^
	14.5	89.92 ± 2.38 ^a^	90.76 ± 1.61 ^a^	85.71 ± 0.84 ^a^
Cineole	4.7	18.80 ± 2.15 ^c^	18.10 ± 2.17 ^c^	19.83 ± 1.65 ^c^
	8	23.93 ± 6.45 ^bc^	25.86 ± 7.78 ^bc^	31.03 ± 9.01 ^bc^
	11.2	35.90 ± 4.27 ^b^	36.21 ± 4.98 ^b^	40.52 ± 3.82 ^b^
	14.5	59.83 ± 1.64 ^a^	56.03 ± 2.17 ^a^	60.34 ± 2.99 ^a^
	17.8	67.52 ± 7.58 ^a^	67.24 ± 8.27 ^a^	65.52 ± 7.84 ^a^

Different lowercase letters indicate significant differences (*p* < 0.05) between concentrations at the same time.

**Table 7:** Contact toxicity of major compounds of EFEO against *S*. *graminum*.

Treatment	Time (h)	Toxicity Regression Curves	Slope	R^2^	LD_50_ (μg/head)	95% CI (μg/head)	*χ* ^2^	*p*	*df*
l-Caryophyllene	4	y = 2.14x − 1.98	2.14 ± 0.27	0.99	8.49	7.45–9.51	3.67	0.299	3
	8	y = 2.18x − 2.11	2.18 ± 0.27	0.98	9.27	8.23–10.36	1.27	0.737	3
	24	y = 1.97x − 1.90	1.97 ± 0.27	0.98	9.16	8.01–10.35	1.28	0.734	3
Lily aldehyde	4	y = 2.37x − 1.26	2.37 ± 0.23	0.98	3.40	3.01–3.79	2.71	0.438	3
	8	y = 2.42x − 1.34	2.42 ± 0.23	0.99	3.57	3.18–3.97	2.03	0.567	3
	24	y = 2.31x − 1.38	2.31 ± 0.24	0.98	3.96	3.52–4.44	4.20	0.240	3
α-Terpineol	4	y = 2.33x − 1.72	2.33 ± 0.19	0.98	5.50	3.71–7.41	7.82	0.050	3
	8	y = 2.33x − 1.76	2.33 ± 0.20	0.99	5.71	3.57–8.11	10.34	0.016	3
	24	y = 2.17x − 1.73	2.17 ± 0.20	0.99	6.27	4.22–8.75	8.14	0.043	3
Cineole	4	y = 2.54x − 2.82	2.54 ± 0.31	0.99	12.88	9.56–22.26	9.12	0.028	3
	8	y = 2.46x − 2.75	2.46 ± 0.31	0.98	13.12	10.34–19.44	5.84	0.120	3
	24	y = 2.28x − 2.48	2.28 ± 0.30	0.99	12.31	11.02–14.00	3.12	0.373	3

**Table 8:** Corrected mortality rate of EFEO against *H. axyridis*.

Time (h)	Corrected Mortality Rate (%)				
	Concentration (μg/head)				
	2.90	5.70	11.50	23.00	45.90
4	00.00 ± 0.00 ^d^	10.00 ± 4.10 ^cd^	20.00 ± 0.00 ^bc^	27.50 ± 4.80 ^b^	52.50 ± 4.80 ^a^
8	00.00 ± 0.00 ^d^	12.50 ± 2.50 ^cd^	20.00 ± 0.00 ^bc^	27.50 ± 4.80 ^b^	62.50 ± 8.50 ^a^
24	12.50 ± 2.50 ^c^	22.50 ± 2.50 ^bc^	25.00 ± 2.50 ^bc^	32.50 ± 4.80 ^b^	65.00 ± 9.60 ^a^
48	22.50 ± 4.80 ^c^	32.50 ± 4.80 ^bc^	32.50 ± 2.50 ^bc^	42.50 ± 8.50 ^b^	67.50 ± 7.50 ^a^

Means in the same row followed by different lowercase letters differed significantly (*p* < 0.05).

**Table 9:** Contact toxicity of EFEO against *H. axyridis*.

Time (h)	Toxicity Regression Curves	Slope	R^2^	LD_50_ (μg/head)	95% CI (μg/head)	*χ* ^2^	*p*	*df*
4	y = 1.75x − 2.89	1.75 ± 0.30	0.97	44.80	32.06–80.17	2.26	0.520	3
8	y = 1.88x − 2.96	1.88 ± 0.34	0.98	37.34	27.97–58.77	5.17	0.180	3
24	y = 1.12x − 1.76	1.12 ± 0.24	0.98	36.71	23.68–83.77	4.99	0.292	3
48	y = 0.86x − 1.22	0.86 ± 0.22	0.97	26.07	15.94–67.68	3.85	0.441	3

**Table 10:** Corrected mortality rate of pyrethrin against *H. axyridis*.

Time (h)	Corrected Mortality Rate (%)				
	Concentration (μg/head)				
	0.0444	0.0666	0.0887	0.1109	0.1331
4	15.00 ± 2.90 ^e^	27.50 ± 2.50 ^d^	47.50 ± 2.50 ^c^	67.50 ± 2.50 ^b^	77.50 ± 2.50 ^a^
8	22.50 ± 4.80 ^d^	40.00 ± 4.10 ^c^	57.50 ± 4.80 ^b^	72.50 ± 2.50 ^a^	80.00 ± 4.10 ^a^
24	30.80 ± 2.60 ^c^	41.00 ± 4.90 ^c^	69.20 ± 4.20 ^b^	82.10 ± 2.60 ^a^	84.60 ± 3.00 ^a^
48	32.40 ± 2.70 ^d^	43.20 ± 2.70 ^c^	70.30 ± 2.70 ^b^	83.80 ± 3.10 ^a^	86.50 ± 2.70 ^a^

Means in the same row followed by different lowercase letters differed significantly (*p* < 0.05).

**Table 11:** Contact toxicity of pyrethrin against *H. axyridis*.

Time (h)	Toxicity Regression Curves	Slope	R^2^	LD_50_ (μg/head)	95% CI (μg/head)	*χ* ^2^	*p*	*df*
4	y = 3.93x + 4.15	3.93 ± 0.61	0.98	0.09	0.08–0.10	0.91	0.822	3
8	y = 3.42x + 3.82	3.42 ± 0.58	0.99	0.08	0.07–0.09	0.19	0.979	3
24	y = 3.55x + 4.18	3.55 ± 0.61	0.97	0.07	0.06–0.08	1.97	0.580	3
48	y = 3.62x + 4.31	3.62 ± 0.65	0.98	0.07	0.05–0.07	1.70	0.637	3

**Table 12:** The population decline rate of *S. graminum* by EFEO nanoemulsion.

Time (d)	Population Decline Rate (%)		
	Treatment		
	EFEO Nanoemulsion	10% EFEO	CK
1	54.00 ± 6.11 ^a^	32.67 ± 6.57 ^b^	0.00 ± 0.00 ^c^
3	73.33 ± 4.80 ^a^	48.00 ± 4.16 ^b^	0.00 ± 0.00 ^c^
5	74.67 ± 4.67 ^a^	60.00 ± 2.31 ^b^	6.00 ± 2.00 ^c^

Means in the same row followed by different lowercase letters differed significantly (*p* < 0.05).

## Data Availability

The original contributions presented in this study are included in the article/Supplementary Materials. Further inquiries can be directed to the corresponding author.

## Supplementary Materials

The following supporting information can be downloaded at https://www.mdpi.com/article/10.3390/insects16111141/s1. Table S1. Chemical composition of EFFO; Table S2. Effects of EFEO LD_50_ on the developmental duration and fecundity of F_1_ and F_2_ of *S. graminum*. Table S3. Effects of EFEO LD_50_ on the vital parameters of F_1_ and F_2_ of *S. graminum*. Figure S1. Fumigation effect of EFEO against *S*. *graminum*, for 24 h. Figure S2. FTIR spectrum of prepared nanoparticles.
