# Supplementary material for: Insecticidal Activity of Eupatorium fortunei Essential Oil Against Schizaphis graminum and Its Effects on Detoxification Enzymes
Source: Insects. 2025 Nov 7;16(11):1141. doi: 10.3390/insects16111141 (PMC12653214; doi:10.3390/insects16111141)
Supplement: Supplementary file 1 [file insects-16-01141-s001.zip › insects-3906403-supplementary.pdf]

Toxicity and transgenerational biological traits effects of *Eupatorium fortune* essential oil on *Schizaphis graminum* (Rondani)

Guochang Wang [1,†], Dongbiao Lü [1,†,*], Xing Ge [1], Ziyue Zhang [1], Fanning Meng [1], Liuping Chen [1], Kassen Kuanysh [2], Xinan Li [1], Baizhong Zhang [1,4], Sarsekova Dani [3,*] and Hongliang Wang [1,*]

1 Henan Province Engineering Research Center of Biological Pesticide & Fertilizer Development and Synergistic Application, College of Plant Protection and Environment, Henan Institute of Science and Technology, Xinxiang 453003, China; wgchslbh@163.com (G.W.); ge_xing_22@126.com (X.G.); zzy_94983@163.com (Z.Z.); luckymfn@163.com (F.M.); chenliupinghist@163.com (L.C.); lixinan2019@126.com (X.L.); baizhongok@163.com (B.Z.)

2 Plant Biotechnology Center, Kazakh National Agrarian Research University, Almaty 050040, Kazakhstan; kassenkuanysh12@gmail.com

3 Hebi Institute of Engineering and Technology, Henan Polytechnic University, Hebi 458030, China

4 Faculty of Forestry and Land Resources, Kazakh National Agrarian Research University, Almaty 050040, Kazakhstan

* Correspondence: dongbiao_0613@163.com (D.L.); dani999@mail.ru (S.D.); wanghlzb@163.com (H.W.)

† These authors contributed equally to this work..

Tables

Table S1. Chemical composition of EFFO.

NO.	Retention time	Chemical compound	Molecular formula	Relative content (%)
1	7.085	1S-alpha-Pinene	$C_{10}H_{16}$	0.60
2	9.226	1S-1-beta-Pinene	$C_{10}H_{16}$	0.07
3	9.695	Myrcene	$C_{10}H_{16}$	0.05
4	10.577	p-mentha-1,5-diene	$C_{10}H_{16}$	0.08
5	11.597	Cyclohexene	$C_{10}H_{16}$	1.29
6	12.396	Cineole	$C_{10}H_{18}O$	10.74
7	13.288	g-Terpinene	$C_{10}H_{16}$	0.19
8	17.965	1-terpineol	$C_{10}H_{18}O$	0.28
9	18.912	1-Methyl-4-(1-methylethenyl)-cyclohexanol	$C_{10}H_{18}O$	1.45
10	20.042	E-β-Terpineol	$C_{10}H_{18}O$	0.67
11	20.309	1-chloro-2-methyl-propan-2-ol	$C_4H_9C_lO$	0.20
12	21.494	α-Terpineol	$C_{10}H_{18}O$	12.17
13	23.516	1-(4-Butylphenyl)-ethan-1-one	$C_{12}H_{16}O$	0.06
14	26.898	Copaene(6CI)	$C_{15}H_{24}$	0.34
15	28.267	4-tert-Butylbenzaldehyde	$C_{11}H_{14}O$	0.17
16	28.809	Bicyclo-(7.2.0)-undec-4-ene,4,11	$C_{15}H_{24}$	0.19
17	28.984	d-Longifolene	$C_{15}H_{24}$	0.22
18	29.912	l-Caryophyllene	$C_{15}H_{24}$	44.66
19	30.427	Benzenemethanol,4-(1,1-dimethylethyl)	$C_{11}H_{16}O$	0.11
20	31.345	1,4,8-Cycloundecatriene,2,6,6,9-tetramethyl	$C_{15}H_{24}$	1.54
21	33.634	pentamethyl phenyl	$C_{11}H_{16}$	0.05
22	34.433	4-t-butyl propiophone	$C_{13}H_{18}O$	0.17
23	36.841	Cyclohexene,3-methyl-6-(1-methylethenyl)	$C_{10}H_{16}$	0.12
24	37.521	Lily aldehyde	$C_{14}H_{20}O$	22.36
25	38.495	4,5-Epoxy-4,11,11-trimethyl-8	$C_{15}H_{24}O$	1.91
26	40.701	Pyridine,2-methyl-5-(1-methylethenyl)	$C_9H_{11}N$	0.10
27	42.915	Phenol,2,6-bis(1,1-dimethylethyl)-4-methyl-,1-(N-methylcarbamate)	$C_{17}H_{27}NO_2$	0.16
28	43.77	Campholenic aldehyde	$C_{10}H_{16}O$	0.06

Table S2. Effects of EFEO LD_{50} on the developmental duration and fecundity of F_1 and F_2 of *S. graminum.*

Development stages	Generation	Developmental periods (day) and Number of offspring (nymphs/per)	
		CK	LD_{50}
1st instar	F_1	1.51±0.08 a	1.52±0.09 a
	F_2	1.37±0.06 a	1.48±0.08 a
2nt instar	F_1	1.42±0.07 a	1.52±0.08 a
	F_2	1.24±0.06 a	1.27±0.06 a
3rd instar	F_1	1.19±0.0 5a	1.22±0.05 a
	F_2	1.17±0.05 a	1.24±0.05 a
4th instar	F_1	1.30±0.06 a	1.35±0.07 a
	F_2	1.52±0.06 a	1.48±0.06 a
Preadult	F_1	5.43±0.09 a	5.51±0.09 a
	F_2	5.32±0.09 a	5.43±0.09 a
Adult longevity	F_1	8.94±0.65 a	10.26±0.65 a
	F_2	9.30±0.58 a	9.44±0.64 a
Total longevity	F_1	12.15±0.76 a	12.11±0.93 a
	F_2	12.67±0.70 a	12.47±0.77 a
APOP	F_1	0	0
	F_2	0.02±0.02 a	0
TPOP	F_1	5.43±0.10 a	5.50±0.10 a
	F_2	5.32±0.09 a	5.43±0.09 a
Aphid production	F_1	8.00±0.56 a	8.91±0.71 a
	F_2	7.95±0.51 a	7.66±0.52 a
Fecundity (nymphs/per)	F_1	45.21±3.11 a	50.43±3.79 a
	F_2	49.57±3.26 a	48.75±3.13 a

APOP: Adult pre-oviposition period. TPOP: Total pre-oviposition period. Means in the same row followed by different lowercase letters differed significantly ($P<0.05$).

Table S3. Effects of EFEO LD_{50} on the vital parameters of F_1 and F_2 of *S. graminum.*

Parameters	Generations	CK	LD_{50}
r_m (day^{-1})	F_1	0.41±0.01 a	0.40±0.01 a
	F_2	0.42±0.01 a	0.42±0.01 a
λ (day^{-1})	F_1	1.50±0.02 a	1.48±0.02 a
	F_2	1.52±0.01 a	1.52±0.01 a
R_0 (offspring individual^{-1})	F_1	36.76±3.25 a	36.78±3.78 a
	F_2	41.64±3.44 a	39.64±3.34 a
T (day)	F_1	8.81±0.16 a	9.17±0.17 a
	F_2	8.84±0.11 a	8.80±0.12 a

Means in the same row followed by different lowercase letters differed significantly ($P<0.05$).

Figures

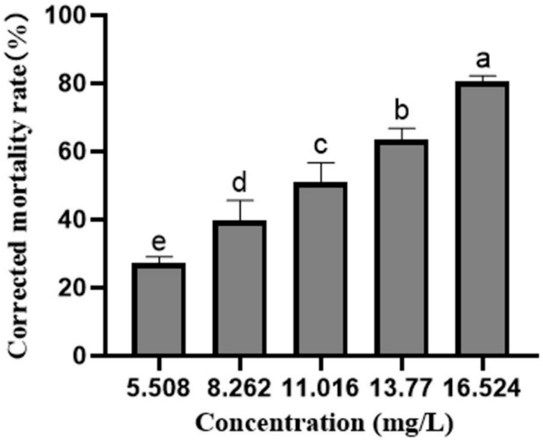

Figure S1. Fumigation effect of EFEO against *S. graminum* for 24 h.

Different letters (a, b, c …) above the bars represent significant differences by ANOVA with Tukey's HSD test (P<0.05).

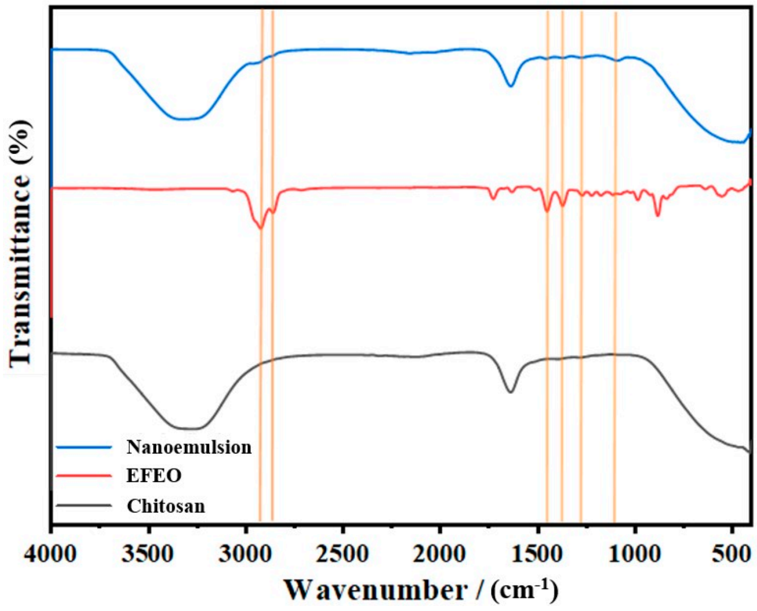

Figure S2. FTIR spectrum of prepared nanoparticles.
